# Performance of compulsive behavior in rats is not a unitary phenomenon – validation of separate functional components in compulsive checking behavior

**DOI:** 10.1111/ejn.12652

**Published:** 2014-06-16

**Authors:** Mark C Tucci, Anna Dvorkin-Gheva, Eric Johnson, Paul Cheon, Leena Taji, Arnav Agarwal, Jane Foster, Henry Szechtman

**Affiliations:** 1 Department of Psychiatry and Behavioural Neurosciences, Health Science Centre, McMaster UniversityRoom 4N82, 1280 Main Street West, Hamilton, ON, Canada, L8S 4K1

**Keywords:** 8-hydroxy-2-(di-n-propylamino) tetralin hydrochloride, analysis and synthesis, animal model, compulsive checking behavior, nucleus accumbens core lesion

## Abstract

A previous analysis of the quinpirole sensitisation rat model of obsessive-compulsive disorder revealed that the behavioral phenotype of compulsive checking consists of three constitutive components – vigor of checking performance, focus on the task of checking, and satiety following a bout of checking. As confirmation of this analysis, the aim of the present study was to reconstitute, without quinpirole treatment, each of the putative components, with the expectation that these would self-assemble into compulsive checking. To reconstitute vigor and satiety, the employed treatment was a bilateral lesion of the nucleus accumbens core (NAc), as this treatment was shown previously to exaggerate these components. To reconstitute focus, the employed treatment was a low dose of the serotonin-1A receptor agonist 8-hydroxy-2-(di-n-propylamino) tetralin hydrochloride (DPAT) (0.0625 mg/kg), as high doses of this drug induce compulsive behavior and exacerbate focus. Results showed that injection of DPAT to NAc lesion rats did yield compulsive checking. Neither the drug alone nor the NAc lesion by itself produced compulsive checking. The demonstrated synthesis of compulsive checking by the combined treatment of low-dose DPAT and NAc lesion strengthened the previous fractionation of the model obsessive-compulsive disorder phenotype into three constitutive components, and suggested a role for serotonin-1A receptors outside the NAc in enhanced focus on the task of checking.

## Introduction

There is a general consensus that the neurobiological substrate underlying obsessive-compulsive disorder (OCD) stems at least in part from a dysfunction that results in persistent neural activity within a series of cascading cortical-basal ganglia-thalamo-cortical loops (Wise & Rapoport, 1989; Saxena *et al*., 1998; Graybiel & Rauch, 2000; Stein, 2002; Aouizerate *et al*., 2004; Huey *et al*., 2008; Szechtman *et al*., 2014), probably involving alterations in serotonin (5-HT) and dopamine (DA) signaling (Goodman *et al*., 1990; Zohar *et al*., 2000; Westenberg *et al*., 2007; Nikolaus *et al*., 2010). However, relatively little is known about the specific behavioral process, or processes, that is altered in this neurocircuit to yield OCD, and more generally, whether the OCD phenotype is the manifestation of a unitary functional system or a collection of constitutive components. To address this question, a recent study employed the quinpirole sensitisation rat model of OCD (Szechtman *et al*., 1998; Eilam & Szechtman, 2005), and revealed that quinpirole-induced compulsive checking behavior is not a unitary phenomenon but consists of three relatively independent components, all greatly exaggerated by quinpirole: (i) vigor of checking; (ii) the focus on checking; and (iii) rest or ‘satiety’ after a bout of checking (Dvorkin *et al*., 2010). This decomposition exposed ‘compulsive’ behavior as highly motivated performance but without apparent satiation (Dvorkin *et al*., 2010), and suggested the possibility of a similar explanation for OCD compulsions, theorised to stem from a dysfunction in ‘the sense of task completion’ (Pitman, 1989), ‘just right feeling’ (Leckman *et al*., 1994; Wahl *et al*., 2008), ‘feeling of incompleteness’ (Rasmussen & Eisen, 1992; Summerfeldt, 2004; Zor *et al*., 2011) or ‘feeling of knowing’ (Szechtman & Woody, 2004; Hinds *et al*., 2012).

The hypothesis that exaggerated function in three elemental processes produces compulsive checking behavior would be strengthened if this model OCD-like phenotype could be ‘synthesised’ experimentally, without quinpirole treatment (Teitelbaum & Pellis, 1992; Teitelbaum, 2012). That is, if each component process could be activated specifically by a non-quinpirole treatment, would the simultaneous activation of all three yield compulsive checking behavior? The present study addresses this question. It builds on the reported findings that, in saline-treated rats, an excitotoxic lesion of the nucleus accumbens core (NAc) increases the vigor of checking and reduces the rest period after a bout of checking, without inducing compulsive checking behavior (Dvorkin *et al*., 2010). That is, NAc lesion does not increase the concentration on the task of checking, and affects only two of the three components needed for compulsive checking. Hence, we hypothesised that a pharmacological treatment that increases the focus on checking would result, in NAc lesion rats, in compulsive checking behavior. Because of the body of evidence suggesting a role for 5-HT in OCD (Barr *et al*., 1993; Zohar *et al*., 2000; Aouizerate *et al*., 2005), we employed as the pharmacological treatment the selective 5-HT_1A_ receptor agonist, 8-hydroxy-2-(di-n-propylamino) tetralin hydrochloride (DPAT). We injected NAc lesion rats with DPAT and examined whether such stimulation would increase focus, yielding compulsive checking behavior. The rationale for the choice of this compound was as follows.

The possible importance of 5-HT_1A_ receptors in OCD is suggested by findings in two animal tests, the marble-burying test (Njung'e & Handley, 1991) and DPAT-induced disruption of spontaneous alternation (Yadin *et al*., 1991). Both of these tests are useful in screening for anti-OCD compounds and have revealed that the positive effects of 5-HT reuptake inhibitors involve activity at the 5-HT_1A_ receptor (Yadin *et al*., 1991; Ichimaru *et al*., 1995). Moreover, like quinpirole, high doses of DPAT can induce compulsive checking behavior (Alkhatib *et al*., 2013) but low doses of DPAT only increase the focus on checking, without inducing compulsive checking (H. Szechtman, unpublished observations). Also of relevance are reports in the literature that attentional focus, which may be related to the present concept of ‘focus on the task of checking’, is enhanced in some behavioral tests by injection of DPAT (Winstanley *et al*., 2003; Carli *et al*., 2005). Hence, we hypothesised that low-dose DPAT would increase focus and add to the NAc lesion effects on vigor and satiety, yielding compulsive checking behavior.

## Materials and methods

### Animals

The subjects were 94 experimentally naive adult male Long Evans rats weighing 250–300 g at the onset of the experiment. Animals were housed in a climate-controlled rat colony room with a 12 h light/dark cycle (lights on at 06:00 h, lights off at 18:00 h). Testing occurred during the light phase. Food and water were freely available. Upon arrival at the animal facility, animals were allowed to acclimatise to the facility for 7 days, followed by handling for 5 days for approximately 2–5 min each day before receiving stereotaxic surgery. Following surgery, rats received approximately 14 days of recovery and during the last 3 days before the start of testing, they were handled daily for 2–5 min as before. Animals were housed and tested in compliance with the regulations set forth by the guidelines of the Canadian Council on Animal Care and approved by the animal Research Ethics Board of McMaster University.

### Drugs

8-Hydroxy-2-(di-n-propylamino) tetralin hydrochloride (Sigma Aldrich, USA) was administered at a dose of 0.0625 mg/kg. This dose was chosen because it increases the focus on checking (H. Szechtman, unpublished observations) and higher doses induce full-blown compulsive checking (Alkhatib *et al*., 2013). The drug was dissolved in 0.9% physiological saline and administered by a subcutaneous injection under the nape of the neck at a volume of 1 mL/kg. Control animals received an equivalent volume of saline in a similar manner.

### Surgery

The excitotoxin, *N*-methyl-d-aspartate (Sigma Aldrich), was dissolved in phosphate-buffered saline at a concentration of 12 mg/mL to produce neurotoxic lesions. For sham lesions, an equivalent volume of phosphate-buffered saline was injected. Intracranial injections of *N*-methyl-d-aspartate and phosphate-buffered saline were made using a 10 μL non-coring Hamilton syringe (Hamilton Company, USA) mounted to a motorised Ultra Micro Pump (World Precision Instruments, USA) that was attached to the arm of a Stereotaxic Apparatus (David Kopf Instruments, USA). Vaporised isoflurane (Pharmaceutical Partners of Canada, Canada) was used to anesthetise the animals, and lidocaine hydrochloride (0.002 mg; Astra Zeneca, Canada) was injected subcutaneously at the surgical site. The post-operative non-steroidal anti-inflammatory analgesic Anafen (0.05 mg/kg; Merial, Canada) was administered subcutaneously at 10 min prior to the end of surgery. The coordinates for the NAc lesion were – Anterior/Posterior, +1.2 mm from bregma; Medial/Lateral, +1.9; Dorsal/Ventral, −7.0 mm from dura. At the injection site, 0.3 μL of the solution was injected bilaterally at a rate of 0.1 μL/min, and the needle was left in place for 5 min to allow the neurotoxin to sufficiently diffuse away from the needle tip.

### Histology

After the final test, rats were killed using carbon dioxide. Brains were removed and flash frozen in −60 °C methylbutane, placed on dry ice for 1 min, wrapped in aluminum foil, and stored in a −80 °C freezer until sectioning. Brains were mounted for sectioning using Tissue-Tek Optimum Cutting Temperature compound and placed in a cryostat for 1 h to thaw to −18 °C. The coronal plane was sectioned at 12 μm thickness, with approximately every ninth section collected on a gelatin-coated slide and stored in a −35 °C freezer until immunohistochemistry. The location and size of lesions were visualised using neuronal nuclei protein immunohistochemistry; sections were stained using monoclonal mouse anti-neuronal nuclei (1 mg/mL; Chemicon International, USA) as the primary antibody, followed by a biotinylated monoclonal anti-mouse IgG (0.5 mg/mL; Vector Laboratories, Canada) as the secondary antibody according to a previously described procedure (Jongen-Relo & Feldon, 2002). Following neuronal nuclei staining, each section was examined for the location and size of the lesion using an Axioskope microscope and Axiovision 4.3 software system (Carl Zeiss Microimaging Inc., USA). Lesion boundaries inside the region of interest (ROI) were demarcated, areas computed, and expressed as a percentage of the ROI area. To compute the ROI lesion area, brain sections at (or nearest to) the pre-determined atlas plates (Paxinos & Watson, 1998) were taken (NAc – plates 12, 14 and 16) and the percent of the ROI lesion at these plates were averaged to obtain the mean percent of the ROI lesion. To be included for behavioral analysis, the minimum lesion size was 55% of the total ROI, following the lesion criterion set in Dvorkin *et al*. (2010).

### Apparatus

Animals were tested on a large open field (160 × 160 cm table without walls) that was located in a non-colony experiment room, as described previously (Dvorkin *et al*., 2006b, 2010). The table was divided virtually into a grid of 25 rectangular places (locales), but no lines were actually marked on the table surface. Four small Plexiglas/glass boxes (approximately 8 × 8 × 7.5 cm) were located at the same fixed location on the open field throughout the experiment; two were located at corners and two were located at places near the center of the open field. After each rat was tested, the table and objects were wiped clean with a diluted solution of an antibacterial cleaner (Lysol). The behavior of animals on the open field was videotaped continuously by a camera affixed to the ceiling (providing a stationary top view of the entire open field and the rat in it). Videotapes were converted to MPEG files (Canopus MPEGPro EMR realtime MPEG-1 MPEG-2 encoder) and these digitised videos were used to automatically track the trajectories of locomotion using EthoVision 3.1 (Noldus Information Technology, Netherlands) software (Noldus *et al*., 2001; Spink *et al*., 2001).

### Data analysis

From the digitised video files, EthoVision 3.1 software was used to extract the time series of *x*,*y* coordinates of the rat in the open field (Dvorkin *et al*., 2006b). To remove noise, digitised tracking data were pre-processed (by applying appropriate filters to smooth the *x*,*y* coordinates; Hen *et al*., 2004), and the obtained coordinates were divided into episodes of forward locomotion (called progression) and episodes of small movements or immobility (called lingering), as described previously (Golani *et al*., 1993; Drai *et al*., 2000; Drai & Golani, 2001). The coordinate system was mapped onto the 25 open field locales (places) (Szechtman *et al*., 1998), and the frequency of visits and duration of stops in each locale were computed (the terms ‘visit’ and ‘stop’ are equivalent and are used interchangeably). Checking behavior was defined with reference to the most-visited locale (labeled ‘key place’ or ‘key locale’; these terms are equivalent), which in most instances was also the locale with the longest total duration of stops (Eilam & Golani, 1989; Szechtman *et al*., 1998). A visit to the key place was also referred to as a ‘check’ or ‘checking’, and the following set of four criteria measures of checking behavior were computed. (i) Frequency of checking – total number of visits to the key locale. (ii) Length of check – total duration of stay at the key locale divided by the frequency of visits there; this measure was also an indirect index of ritual-like behavior as the appearance of motor rituals in quinpirole-treated rats is associated with a very short duration of stay in the key locale (Szechtman *et al*., 1998; Ben Pazi *et al*., 2001). (iii) Recurrence time of checking – mean duration of return times to the key locale (‘return time’ is the interval from departure to next arrival at the locale). (iv) Stops before returning to check – mean number of places visited between returns to the key locale. Compulsive checking behavior was identified by the presence of a significant difference compared with saline-treated control animals; all four measures needed to differ from controls for the claim of compulsive checking (Szechtman *et al*., 1998), and hence the group of these four measures was termed ‘criteria measures’ for compulsive checking.

The profile of compulsive checking behavior has been dissociated into a set of functional components in a lesion study (Dvorkin *et al*., 2010). Specifically, a lesion to the NAc altered the amount of checking behavior (as indexed by a change simultaneously in both the ‘frequency of checking’ and ‘length of check’), whereas a lesion to the orbital frontal cortex affected the delay between checks of the key locale (as indexed by a change simultaneously in both the ‘time to return to check’ and ‘number of stops before returning to check’). This pattern of results suggested that the functional roles of the NAc and orbital frontal cortex in checking behavior are to control the vigor of motor performance and the focus on goal-directed activity, respectively (Dvorkin *et al*., 2010). Accordingly, we considered vigor and focus as two relatively independent components of checking behavior, where a change in the vigor of checking was indexed by concurrent changes in the ‘frequency of checking’ and ‘length of check’, and similarly where a change in the focus on checking was indexed by concurrent changes in the ‘time to return to check’ and ‘number of stops before returning to check’.

In addition to the above criteria measures, the ‘time to next checking bout’ was also evaluated (Dvorkin *et al*., 2006b). This measure is greatly reduced in quinpirole-sensitised rats showing compulsive checking, and has been proposed to index the third constitutive component of compulsive checking behavior, i.e. ‘satiety’ or rest after checking (Dvorkin *et al*., 2010). It was reasoned that, in the animal model, the reduced ‘satiety’ or ‘rest’ after a bout of checking corresponded to notions that OCD reflects failure to achieve a ‘sense of task completion’ (Pitman, 1989) or ‘feeling of knowing’ (Szechtman & Woody, 2004). The computation of checking bouts is detailed in Dvorkin *et al*. (2006b). Briefly, the method followed the logic used to identify the clustering of a bout of eating behavior into a ‘meal’ and the time between meals into a period of post-ingestion satiety (Tolkamp *et al*., 1998; Tolkamp & Kyriazakis, 1999). A bout of behavior, according to those authors, is defined on the basis of the distribution of time intervals between behavioral events (interevent intervals). This distribution is examined to locate and extract a time-point that will produce a natural split between clusters of interevent intervals. Specifically, the identified time-point will separate the time intervals into a class of (relatively long) intervals that are between the bouts of behavior (interbout intervals) and a class of (relatively shorter) intervals that belong within a bout of behavior (intrabout intervals; Tolkamp *et al*., 1998; Tolkamp & Kyriazakis, 1999). This principle was employed in an algorithm developed to identify bouts of checking behavior and extract the ‘time to next checking bout’ (Dvorkin *et al*., 2006b). A rat may complete a bout of checking but not start the next bout during the session and hence the number of rats used for the ‘time to next checking bout’ was generally smaller than for the four criteria measures for compulsive checking. Generally, saline-treated rats had 1–2 bouts of checking behavior in a session, whereas quinpirole-treated rats usually performed two or more bouts (Dvorkin *et al*., 2006b, 2010). Following the procedure modified in Tucci *et al*. (2013), if more than one bout of checking was performed, only the first ‘time to next checking bout’ was used for statistical analysis.

### Design and procedure

The study consisted of a 2 × 2 fully crossed factorial design where one of the between-group factors was *Lesion* (sham lesion vs. NAc lesion) and the other was *Drug* (saline vs. DPAT). Animals were assigned to treatment groups at 2–3 days before the start of behavioral testing based on body weight.

Following recovery from surgery, testing on the open field began. Rats were weighed in the colony room and transported to the experiment room containing the open field. Rats were administered their assigned treatment appropriately, and immediately gently placed onto the center of the open field. Each trial lasted 55 min. Rats received a total of four trials, each separated by 2–3 days. After the final trial, rats were killed and brains were collected as described above.

### Statistical analysis

The research question of the present study was whether two treatments had an additive effect and more specifically whether those treatments, which on their own did not yield compulsive checking, did so when combined. Hence, the experiment tested the hypothesis that the combination of an NAc lesion and a serotonergic agonist (DPAT) together will yield compulsive checking but each treatment separately will not. A *Lesion* (sham lesion vs. NAc lesion) by *Drug* (saline vs. DPAT) anova was used to evaluate each criterion measure of compulsive checking and the measure of post-checking satiety. Compulsive checking was defined by a significant difference from saline-treated controls on all criteria measures. It was expected that NAc lesion would increase vigor, demonstrated by a significant main effect of lesion on the two vigor-related measures (frequency of checking and length of check) and no significant effects of lesion on the two focus-related measures (recurrence time of checking and stops before returning to check). Similarly, it was expected that DPAT would increase focus, demonstrated by a significant main effect of drug for the measures of focus and no significant effects of drug on the measures of vigor. Accordingly, it was expected that compulsive checking behavior would be evident only in the NAc lesion group injected with DPAT because only in this group would there be an effect on frequency of checking and length of check (due to a main effect of lesion), and on the recurrence time of checking and stops before returning to check (due to a main effect of drug). In addition, it was expected that the NAc lesion group injected with DPAT would show a reduced time to the next checking bout due to a main effect of lesion. The significance level was set at *P *<* *0.05. Analysis was computed using spss 20 for Windows. Values presented in graphs are the mean and SEM.

We analysed and present data for the fourth trial only. It was reasoned that this test would be free of any non-specific effects of surgery and should reflect the most stable effects of the drug treatment, as previous observations from our laboratory suggested robust effects around this period (Alkhatib *et al*., 2013). A total of six tests across four groups had missing data for the fourth trial and for these rats trial 3 was used instead.

## Results

### Histology

**Figure 1:**
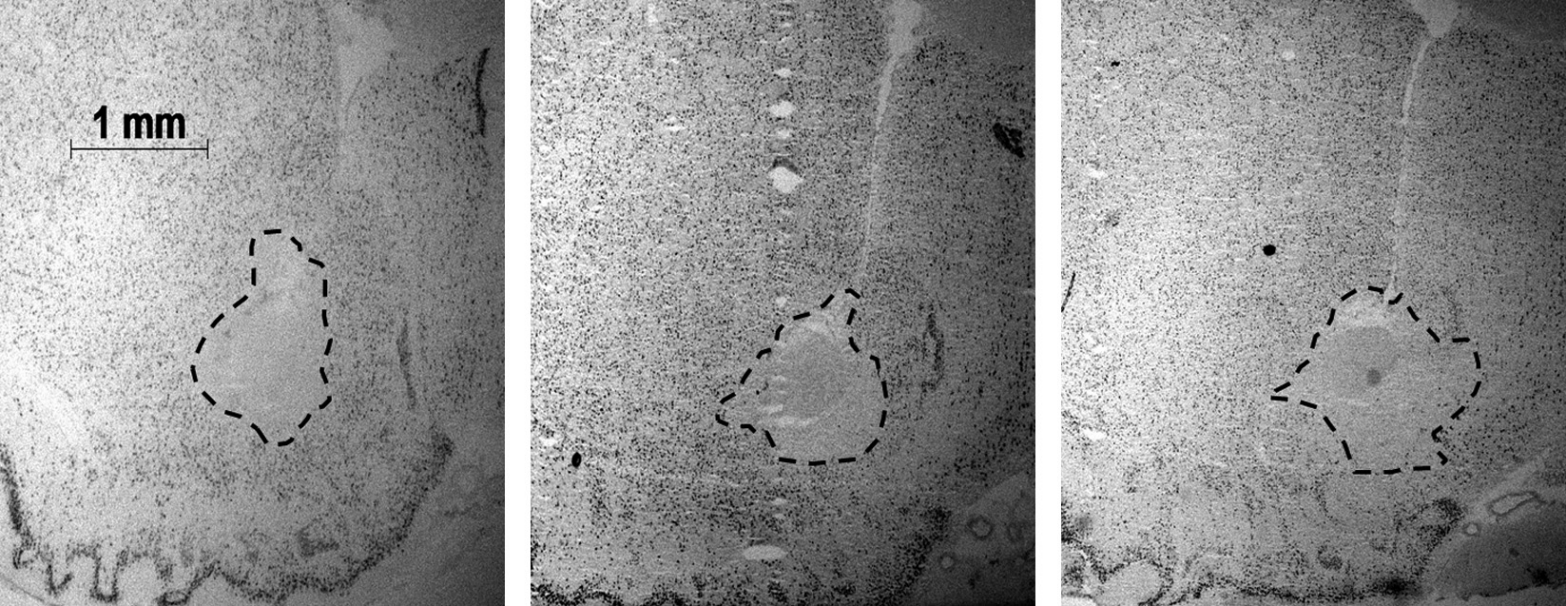
A representative neuronal nuclei-stained section for NAc lesion in a rat representative of the average cell-body damage across lesion groups. The left, middle and right panels represent atlas plates 12, 14 and 16 across the NAc, respectively (Paxinos & Watson, 1998). These plates are located 2.76, 2.28, and 2.04 mm from bregma, respectively. The dashed line demarcates the area of cell damage.

Animals that met the criterion of at least 55% lesion to the NAc were included in the behavioral analysis (Dvorkin *et al*., 2010). The number of animals for each treatment condition is shown in Table1. The average size of the cell-body lesion across the NAc lesion groups was approximately 74% of the total ROI (Table1). Figure1 shows a representative NAc lesion animal with size of cell-body destruction similar to the mean across NAc lesion groups. Across atlas plates 12, 14 and 16 (Paxinos & Watson, 1998), lesions were well localised within the accumbens core subregion, with minimal encroachment to the accumbens shell subregion and ventral pallidum at more posterior sections. Sham lesion rats had no detectable damage to the NAc and this is indicated by 0% lesion in Table1.

**Table 1:** Number of rats in each group with proper lesion in the target ROI, NAc, and the percentage of the NAc area that had a lesion (mean ± SEM and the minimum size of a lesion in the group)

	Type of lesion				
	Sham lesion		NAc lesion		
Drug	*N*	Mean (%)	*N*	Mean ± SEM (%)	Minimum (%)
Saline	37	0	25	73.6 ± 0.0	55
DPAT	18	0	14	75.6 ± 0.0	66

### Routes of travel

**Figure 2:**
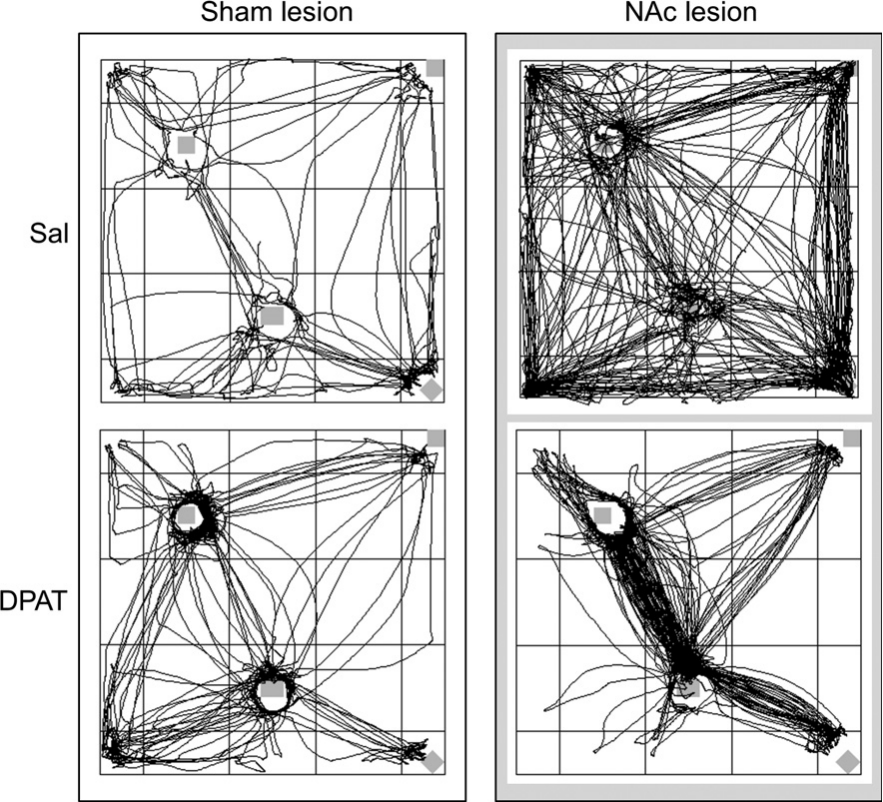
Effects of DPAT on the routes of travel in sham and NAc lesion rats. The routes of travel are shown as path plots for a representative rat with a sham lesion (left column) and NAc lesion (right column) that was treated with saline (Sal) (top row) or DPAT (bottom row); the selected rat has a distance of travel value closest to the group mean. Locomotor trajectories during the entire 55 min session are shown, and each line represents a trajectory of locomotion; the density of trajectory lines corresponds to the amount of locomotion. Gray squares indicate locations of the four objects in the open field.

**Figure 3:**
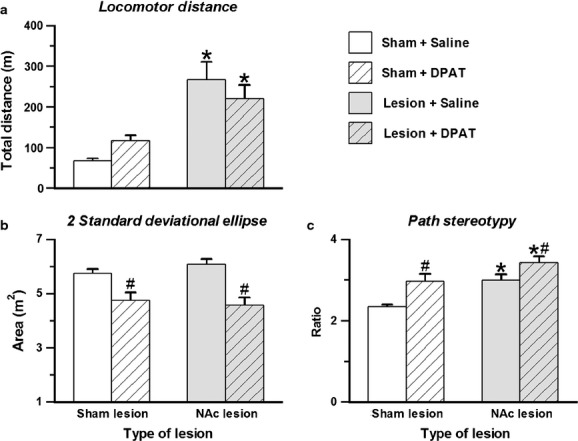
Effects of DPAT on distance traveled (a), two standard deviational ellipse (b) and path stereotypy (c) in sham and NAc lesion rats. Bar graphs show mean performance (+ SEM) in 55 min by each group. Open bars, sham controls injected with saline; hatched bars, sham controls injected with DPAT; gray bars, NAc lesion rats injected with saline; gray hatched bars, NAc lesion rats injected with DPAT. *Main effect of lesion; ^#^main effect of drug.

An overview of the key finding can be gained from a visual inspection of the routes of travel shown in Fig.2. The left column displays paths of locomotion of a typical sham lesion rat treated with saline or DPAT, whereas the right column shows these paths for a corresponding NAc lesion rat. A visual comparison of the routes shown by the different groups makes it evident that an injection of DPAT to the NAc lesion rat produced routes of travel extremely reminiscent of the paths of travel shown by rats treated with quinpirole (see, e.g. Fig. 5 in Dvorkin *et al*., 2010). The quantitative attributes of the paths of locomotion shown by quinpirole rats were: (i) the amount of locomotion was elevated, as measured by the distance traveled; (ii) the spatial extent of the explored space was constricted, as measured by two standard deviational ellipse; and (iii) the paths of locomotion were restricted to traveling along a few routes only, as measured by path stereotypy (Eilam *et al*., 1989; Szechtman *et al*., 1994; Dvorkin *et al*., 2006a,b, 2010). The values for these measures in the present study are shown in Fig.3, and their statistical analysis is presented below.

As shown in Fig.3a, the amount of locomotion was elevated by NAc lesion (main effect of lesion – *F*_1,90_ = 28.39, *P *<* *0.001, 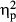 = 0.240) but not by treatment with DPAT (main effect of drug – *F*_1,90_ = 0.002, *P *=* *0.961, 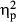 = 0; lesion by drug interaction – *F*_1,90_ = 2.72, *P *=* *0.102, 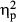 = 0.029). In contrast, as shown in Fig.3b, the spatial extent of exploration was reduced by injection of DPAT (main effect of drug – *F*_1,90_ = 27.56, *P *<* *0.001, 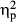 = 0.234) and was not affected by NAc lesion (main effect of lesion – *F*_1,90_ = 0.13, *P *=* *0.723, 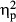 = 0.001; lesion by drug interaction – *F*_1,90_ = 1.15, *P *=* *0.286, 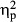 = 0.013). Finally, as shown in Fig.3c, the restriction of hyper-locomotion to a few routes, as demonstrated by increased path stereotypy, was produced by the additive effects of NAc lesion and DPAT (main effect of lesion – *F*_1,90_ = 19.83, *P *<* *0.001, 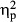 = 0.181; main effect of drug – *F*_1,90_ = 17.89, *P *<* *0.001, 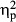 = 0.166; lesion by drug interaction – *F*_1,90_ = 0.65, *P *=* *0.421, 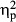 = 0.007).

Thus, the results showed that NAc lesions had a predominant effect on the amount of motor activity, whereas the predominant effects of DPAT were on a process related to the distribution or the focus of this activity. Together, these effects yielded a pattern of locomotor activity that was similar to the locomotor profile of quinpirole-treated rats, suggesting that the quinpirole-like appearance of the routes of travel in the NAc lesion rat injected with DPAT was produced by the additive effects of NAc lesion and DPAT.

### Additive effects of nucleus accumbens core lesion and 8-hydroxy-2-(di-n-propylamino) tetralin hydrochloride on compulsive checking

**Figure 4:**
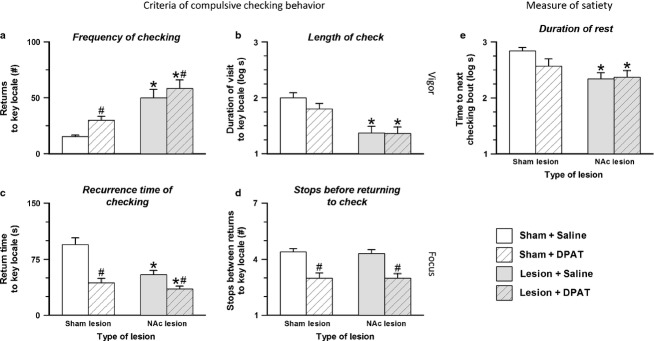
Performance on criteria measures for compulsive checking behavior (a–d) and post-checking rest (e) shown by groups of sham controls and NAc lesion rats treated with saline or DPAT. Open bars, sham controls injected with saline; hatched bars, sham controls injected with DPAT; gray bars, NAc lesion rats injected with saline; gray hatched bars, NAc lesion rats injected with DPAT. *Main effect of lesion; ^#^main effect of drug.

Figure4 displays criteria measures for compulsive checking behavior in groups of rats treated with a lesion of the NAc, an injection of DPAT, or the combination of the two treatments. As shown, there was a main effect of lesion for the two measures of vigor (frequency of checking – *F*_1,90_ = 35.80, *P *<* *0.001, 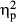 = 0.285; length of check – *F*_1,90_ = 21.18, *P *<* *0.001, 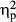 = 0.190) and for the measure of rest (time to next checking bout – *F*_1,50_ = 9.12, *P *=* *0.004, 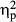 = 0.154). For the two measures of focus, there was a significant but small main effect of lesion for one of the measures (recurrence time of checking – *F*_1,90_ = 8.12, *P *=* *0.005, 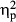 = 0.083) but no significant main effect of lesion for the other measure of focus (stops before returning to check – *F*_1,90_ = 0.40, *P *=* *0.841, 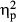 = 0).

As expected, the main effect of drug was significant for the two measures of focus (recurrence time of checking – *F*_1,90_ = 17.11, *P *<* *0.001, 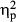 = 0.160; stops before returning to check – *F*_1,90_ = 33.58, *P *<* *0.001, 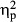 = 0.272). For the two measures of vigor, there was a significant but small main effect of drug for one of the measures (frequency of checking – *F*_1,90_ = 4.94, *P *=* *0.029, 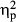 = 0.052) but no significant main effect of drug for the other measure of vigor (length of check – *F*_1,90_ = 0.92, *P *=* *0.341, 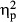 = 0.010). Moreover, as expected, there was no main effect of drug for the measure of rest (time to next checking bout – *F*_1,50_ = 1.11, *P *=* *0.296, 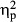 = 0.022).

There was no significant interaction between lesion and drug for any of the measures in Fig.4.

Thus, administration of a low dose of DPAT (0.0625 mg/kg) to NAc lesion rats produced a statistically significant difference between this group and sham + saline controls on all criteria measures for compulsive checking (frequency of checking, length of check, recurrence time of checking, and stops before returning to check) and for the measure of post-checking satiety (Fig.4). Hence, an injection of DPAT to rats with NAc lesion induced full-blown compulsive checking, and only the combination of these two treatments produced this phenomenon.

### Correlations amongst measures of vigor and focus

In a previous study, vigor and focus were identified as two relatively independent components of checking behavior, where a change in the vigor of checking was evident by concurrent effects on both ‘frequency of checking’ and ‘length of check’, and similarly where a change in the focus on checking was manifested by concurrent effects on both the ‘time to return to check’ and ‘number of stops before returning to check’ (Dvorkin *et al*., 2010). However, in the present study, NAc lesion and DPAT showed not only the expected impact on the domains of vigor and focus, respectively, but also produced a small secondary change in one of the measures from the other domain (‘recurrence time for checking’ for NAc lesion and ‘frequency of checking’ for DPAT; Fig.4c and a, respectively).

To shed some light on these findings, Table2 presents the Pearson correlation coefficients between pairs of the four criteria measures. The shaded cells show the correlation for measures within a domain and the remaining cells show correlations between vigor and focus measures. The correlation coefficient for frequency of checking with length of check was very strong and higher than any correlation of these measures with a measure of focus, consistent with the notion that frequency of checking and length of check reflect vigor. Similarly, for the measures of focus (recurrence time of checking and number of stops before returning to check), their correlation coefficient was strong and higher than any correlation with a measure of vigor, except for one correlation, i.e. recurrence time of checking with frequency of checking, which was somewhat stronger than the correlation of the within-focus measures (0.488 vs. 0.410). This latter finding complicated a straightforward interpretation of the pattern of correlations in Table2. If measures of vigor and focus indexed independent components, then the two measures of one domain (e.g. vigor) should correlate with each other more strongly than with the two measures of the other domain (here, with measures of focus). Hence, the observation that the magnitude of the correlation for the two measures of focus did not exceed one of the four between-domain correlations (recurrence time of checking with frequency of checking) cast doubt on vigor and focus being necessarily independent. It suggested instead ‘cross-talk’ between aspects of the focus and vigor components of compulsive checking.

**Table 2:** Pearson correlations between pairs of criteria measures of compulsive checking

	Length of check	Recurrence time of checking	No. of stops before returning to check
Frequency of checking	−0.752**	−0.488**	−0.091
Length of check		0.284**	−0.105
Recurrence time of checking			0.410**

Cells with dark gray color refer to measures within the domain of vigor and cells with light gray color refer to measures within the domain of focus; non-shaded cells show correlations between these domains. The Pearson correlation coefficient was computed for the four groups combined (*N *=* *94).

** *P*≤0.01 (two-tailed).

## Discussion

Animal models of psychopathology can be useful preparations to discover the psychological components of a psychiatric disorder and their underlying neurobiology (Szechtman & Eilam, 2005). An ever richer armament of neuroscience tools can be employed to fractionate the nervous system and thereby split the apparent unity of a phenomenon into its constitutive behavioral components (Teitelbaum & Pellis, 1992; Teitelbaum, 2012). However, the soundness of the findings from such analysis must be confirmed by ‘resynthesis’ (Teitelbaum & Pellis, 1992), i.e. the behavioral components identified in analysis must be ‘put together’ or synthesised into the original phenomenon to demonstrate the correctness of the analysis. This approach of ‘analysis followed by synthesis’ underlies the rationale for the current study where we aimed to synthesise compulsive checking behavior from the components identified in a previous analysis (Dvorkin *et al*., 2010). That analysis of the quinpirole sensitisation rat model of OCD reported that the model compulsive behavior consisted of at least three partially independent processes, all greatly exaggerated by quinpirole – vigor of checking performance, focus on the task of checking, and satiety following a bout of checking (Dvorkin *et al*., 2010). Here, we showed that, without quinpirole, compulsive checking behavior is reconstituted from the simultaneous effects of bilateral lesions to the NAc (which exaggerated predominantly the vigor of checking performance and satiety) and treatment with DPAT (which increased predominantly the focus on the task of checking). This successful synthesis of quinpirole-like compulsive checking behavior by a non-quinpirole method suggests relatively strongly that the previously identified three constitutive components do indeed underlie OCD behavior in the quinpirole sensitisation rat model.

### Relationship between vigor and focus

The combined effects of NAc lesion and DPAT reconstituted not only the quinpirole profile on compulsive checking but also on locomotion; NAc lesion rats injected with DPAT showed more locomotion compared with saline controls but their routes of travel were constricted to a smaller portion of the environment and constrained to relatively few pathways (see Figs2 and 3), like quinpirole-treated rats (Eilam *et al*., 1989; Szechtman *et al*., 1994; Dvorkin *et al*., 2006a,b, 2010). This locomotor pattern (as measured by distance traveled, two standard deviational ellipse, and path stereotypy) is presumably another view on the product of vigor and focus constituting compulsive checking. Accordingly, the increase in the amount of locomotion produced by NAc lesion is consistent with this measure reflecting vigor, whereas the shrinkage in two standard deviational ellipse produced by DPAT is consistent with this measure being related to focus. Interestingly, path stereotypy was increased by NAc lesion and by DPAT and consequently this measure was highest in NAc lesion rats injected with DPAT, the group that showed compulsive checking and quinpirole-like path plots. These findings provide another type of evidence that increases in both vigor and focus are required to produce the quinpirole-like pattern of routes associated with compulsive checking. However, they also suggest that path stereotypy is impacted by both vigor and focus and that this measure is not an exclusive index of either domain.

A similar interpretation may apply to the observation that, in the present study, NAc lesion and DPAT produced a small change in one of the measures from the other domain (‘recurrence time for checking’ for NAc lesion and ‘frequency of checking’ for DPAT; Fig.4c and a, respectively). There are three possible explanations for the observed partial ‘spill-over’ into a second domain: (i) the NAc lesion and DPAT treatments, rather than having effects exclusively on one domain, may also partially impact the other domain; (ii) ‘recurrence time for checking’ and ‘frequency of checking’, rather than being exclusive indices of vigor and focus respectively, may also be open to some modulation by the other domain; or (iii) some combination of both the foregoing alternatives. Although the present study does not truly discriminate amongst these alternatives, there is merit in the second possibility given the relatively high correlation between ‘recurrence time for checking’ and ‘frequency of checking’, and evidence that another measure, path stereotypy, is impacted by both vigor and focus. Nevertheless, regardless of which alternative is correct, none of them discounts the main finding of the present study, which is that to reconstitute compulsive checking two distinct concurrent treatments were necessary – a lesion to the NAc and an injection of DPAT.

### Contribution of dopamine and serotonin receptor subtypes to focus on the task of checking

There are at least three possible neurochemical explanations for the similarity in effects on focus produced by quinpirole in intact rats and DPAT in NAc lesion animals.

1. The focus component is controlled by both DA and 5-HT neurotransmission and involves D2/D3 and 5-HT_1A_ receptors, respectively. Accordingly, focus may be heightened either by D2/D3 stimulation by quinpirole or 5-HT_1A_ activation with DPAT.
2. The focus component is controlled by one or both of the above neurotransmitter systems but quinpirole and DPAT exert their pharmacological activity on the same receptor (D2/D3 or 5-HT_1A_).
3. The focus component has another unidentified target receptor activated equally well by quinpirole and DPAT.

The present study does not discriminate amongst those alternatives and the literature does not discount any of them either. A similar pharmacological profile for quinpirole and DPAT at D2/D3 receptors (Smith & Cutts, 1990; van Wijngaarden *et al*., 1990; Matuszewich *et al*., 1999; Newman-Tancredi *et al*., 1999; Rinken *et al*., 1999) or at 5-HT_1A_ receptors (Ahlenius & Larsson, 1997) had been noted in the literature as a possible explanation for the observed similarity in functional effects, especially as both drugs are ergot derivatives (Ahlenius & Larsson, 1997) although non-ergot compounds that act as an agonist at both D2/D3 and 5-HT_1A_ receptors have been recently synthesised (Glennon *et al*., 2006). However, other studies build on the rich network of neuroanatomical and neurochemical interconnections between 5-HT and DA systems (Barnes & Sharp, 1999; Leger *et al*., 2001; Fink & Göthert, 2007; Filip & Bader, 2009; Albert & Le François, 2010; Hayes & Greenshaw, 2011; Navailles & De Deurwaerdere, 2011) and propose instead that the similarity in function is the outcome of separate regulations via pre-synaptic and/or post-synaptic D2/D3 and 5-HT_1A_ receptors of the interconnected systems (Ahlenius & Larsson, 1997; Matuszewich *et al*., 1999; Clément *et al*., 2006; Shin *et al*., 2012), a schema also proposed for the regulation of the OCD network (Goodman *et al*., 1990; Zohar *et al*., 2000; Szechtman & Woody, 2004; Westenberg *et al*., 2007; Nikolaus *et al*., 2010; Alkhatib *et al*., 2013). Nevertheless, specific experiments are needed to identify whether the focus on the checking component is truly mediated by both D2/D3 and 5-HT_1A_ receptors or only one of them, or conceivably yet another target of quinpirole and DPAT.

Regardless of their identity, it is likely that the pertinent receptors are outside the NAc, given that enhanced focus was produced by DPAT in rats with a lesion of the NAc and the lesion by itself did not enhance focus. Interestingly, a similar finding was observed in the spontaneous alternation paradigm, where the effects of DPAT were enhanced in rats with an electrolytic lesion of the nucleus accumbens, but on its own, the lesion did not affect spontaneous alternations (van Kuyck *et al*., 2003).

### Dopamine and serotonin regulating behavioral components of obsessive-compulsive disorder behavior

Much research implicates a role for both DA and 5-HT in OCD (Goodman *et al*., 1990; Zohar *et al*., 2000; Westenberg *et al*., 2007; Nikolaus *et al*., 2010). In the quinpirole sensitisation rat model of OCD, the model compulsive behavior is induced by repeated injections of quinpirole that result in sensitisation of DA receptors (Szechtman *et al*., 1998, 1999). Hence, the model OCD behavior is driven by sensitised DA activity. However, the present synthesis suggests that the model OCD phenotype also includes activity in parts of the 5-HT systems. Specifically, the enhancement of focus with low-dose DPAT suggests that stimulation of 5-HT_1A_ receptors outside the NAc may serve to enhance compulsive behavior. Other studies in the quinpirole model show normalisation of vigor and post-checking rest with 1-(3-chlorophenyl)-piperazine hydrochloride (Tucci *et al*., 2013, 2014). The authors interpret those studies as suggesting that stimulation of 5-HT_2_ receptors may have a dampening effect on DA activity and promote a reduction of compulsive behavior. Considering the complexity of 5-HT receptors, with pre-synaptic and post-synaptic receptors often having opposite functional effects on 5-HT activity (Barnes & Sharp, 1999; Hoyer *et al*., 2002; Fink & Göthert, 2007; Navailles & De Deurwaerdere, 2011), it is likely that, depending on the behavioral component, either an increase or a decrease in 5-HT activity may promote or ameliorate compulsive behavior. By the same token, the present findings strongly support the current clinical attempts of targeting simultaneously DA and 5-HT activity as a viable pharmacotherapeutic strategy for OCD (Greist *et al*., 2003; Dougherty *et al*., 2004; Koran, 2004; Marazziti *et al*., 2004; Bloch *et al*., 2006).

## Conflict of interest

The authors declare no conflict of interest.

## Abbreviations

5-HT: serotonin
DA: dopamine
DPAT: 8-hydroxy-2-(di-n-propylamino) tetralin hydrochloride
NAc: nucleus accumbens core
OCD: obsessive-compulsive disorder
ROI: region of interest
